# Supplementary material for: Causal Relationship between Aging and Anorexia Nervosa: A White-Matter-Microstructure-Mediated Mendelian Randomization Analysis
Source: Biomedicines. 2024 Aug 16;12(8):1874. doi: 10.3390/biomedicines12081874 (PMC11351342; doi:10.3390/biomedicines12081874)
Supplement: Supplementary file 1 [file biomedicines-12-01874-s001.zip › Supplementary Figure/Figure S8/Leaveoneout plot of MR analysis for TL to ubm-b-1528.pdf]

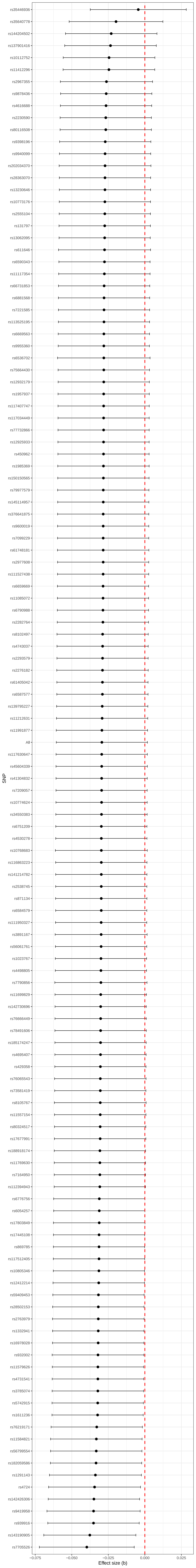
rs35446936
rs35640778
rs144204502
rs137901416
rs10112752
rs11412296
rs2967355
rs9878436
rs4616688
rs2230590
rs80116508
rs9398196
rs9940099
rs202034370
rs28363070
rs13230646
rs10773176
rs2555104
rs131797
rs13062095
rs611646
rs6590343
rs11117354
rs66731853
rs6881568
rs7221585
rs113525195
rs6669563
rs9955360
rs6536702
rs75664430
rs12932179
rs1957937
rs117407747
rs117034449
rs77732866
rs12925933
rs450962
rs1985369
rs150150565
rs79977579
rs145114957
rs376641875
rs9600019
rs7099229
rs61748181
rs2977608
rs111527438
rs6659669
rs11085072
rs6790988
rs2282764
rs8102497
rs4743037
rs2293579
rs2276182
rs61405042
rs6587577
rs139795227
rs11212631
rs11991877
All
rs117630647
rs45604339
rs41304832
rs7209057
rs10774624
rs34550383
rs6751209
rs4530278
rs10768683
rs116863223
rs141214782
rs2538745
rs871134
rs6584579
rs111950327
rs3891167
rs56061761
rs1023767
rs4498805
rs7790856
rs11699829
rs142730698
rs76666449
rs78491606
rs185174247
rs4695407
rs429358
rs76065543
rs73581419
rs8105767
rs11557154
rs80324517
rs17677991
rs188918174
rs11769630
rs7164950
rs112394943
rs6776756
rs6054257
rs17803849
rs17445108
rs869785
rs117512405
rs10805346
rs12412214
rs59409453
rs28502153
rs2763979
rs1332941
rs16978028
rs932002
rs11579626
rs4731541
rs3785074
rs5742915
rs1611236
rs76219171
rs11584821
rs56799554
rs182059586
rs1291143
rs4724
rs142426306
rs9419958
rs939916
rs143190905
rs7705526
SNP
-0.075
-0.050
-0.025
0.000
0.025
Effect size (b)
